# Wnt-Signaling-Mediated Antiosteoporotic Activity of Porcine Placenta Hydrolysates in Ovariectomized Rats

**DOI:** 10.1155/2012/367698

**Published:** 2012-12-03

**Authors:** Byoung-Seob Ko, Da Sol Kim, Suna Kang, Na Ra Lee, Jin Ah Ryuk, Sunmin Park

**Affiliations:** ^1^Korea Institute of Oriental Medicine, Daejeon 305811, Republic of Korea; ^2^Department of Food and Nutrition, Institutes of Basic Sciences, Hoseo University, Asan 336795, Republic of Korea

## Abstract

Anti-osteoporotic effects of two types of porcine placenta hydrolysates (PPH) were evaluated in ovariectomized (OVX) rats orally administered PPH without (WPPH) or with (NPPH) ovarian hormones (1 g/kg bw/day). PPH groups were compared with OVX rats with estrogen replacement (0.1 mg/kg bw conjugated estrogen; EST), or dextrose (placebo; OVX-control) All rats received high-fat/calcium-deficient diets for 12 weeks. NPPH contained less estrogen and progesterone, but more essential amino acids, whereas the opposite was true for WPPH. NPPH decreased body weight and peri-uterine fat pads, and maintained uterus weight. NPPH rats had higher femur and lumbar spine bone mass density compared to controls; but less than those of EST rats. Serum phosphorus and urinary calcium and phosphorus levels were reduced in NPPH rats compared to OVX-controls. Serum bone-specific alkaline phosphatase, osteocalcin, and bone turnover marker levels were reduced NPPH rats compared to OVX-controls. WPPH produced results similar to those of NPPH, but less significant. Both NPPH and estrogen upregulated low-density lipoprotein receptor-related protein 5 and **β**-catenin in OVX rats, while the expression of dickkopf-related protein 1 was suppressed. In conclusion, NPPH exerted anti-osteoporotic effects by activating osteogenesis and stimulating Wnt signaling, possibly mediated by the various amino acids and not ovarian hormones.

## 1. Introduction

Postmenopausal osteoporosis is characterized by a net bone loss due to an imbalance between osteoclast-mediated bone resorption and osteoblast-mediated bone formation [1]. Osteoporosis increases susceptibility to bone fractures and reduces quality of life [1]. Bone mineral density (BMD), a major determinant of osteoporosis, peaks during the early 20 s, after which it decreases continuously at a slow rate and is significantly reduced after menopause [1, 2]. Hormone replacement therapy (HRT) has proven to be efficacious in preventing bone loss and reducing the incidence of skeletal fractures in postmenopausal women [3] but has several side effects that reduce its popularity [4]. However, remedies other than HRT have been used to prevent and treat postmenopausal osteoporosis [5–7]. For example, administration of placenta extracts or hydrolysates from human or other animals by oral or subcutaneous injection [6, 8] has been used to treat osteoporosis.

Treatment with 17*β*-estradiol achieves its positive bone effects through two key actions, facilitating vitamin D-related intestinal calcium absorption [9] and suppressing bone resorption by decreasing the osteoprotegerin/receptor activator of nuclear factor kappa-B l (RANK)/receptor activator of nuclear factor kappa-B ligand (RANKL) system via the Wnt/*β*-catenin signaling pathways [10]. Furthermore, defective Wnt signaling plays a major role in osteoporosis [11]. Premenopausal women with amenorrhea have lower 17*β*-estradiol levels and reduced BMD [12]. In addition, combination treatment with estrogen and progesterone has a more positive effect on BMD than does estrogen alone [13]. Thus, BMD may be increased by HRT, and it is a suitable treatment for preventing bone loss. However, HRT has an adverse effect such as the induction of breast cancer. Researchers have investigated herbs that contain estrogen-like compounds for preventing bone loss [14–17]. Placenta products may be potential substitutes of HRT for preventing osteoporosis. Placental estrogen and progesterone may be effective components if these hormones are bioavailable in placenta hydrolysates and/or extracts. 

The placenta contains various nutrients and hormones that can improve the health of both fetuses and adults and can alleviate postmenopausal symptoms [6, 8]. *Hominis* placenta, dried human placenta, is used as one of several components of traditional remedies such as Yukmi-Jihangtang-Jahage and Honghwain-Jahage, which are used to treat inflammation, hyperlipemia, arteriosclerosis, and gynecological diseases such as osteoporosis and bone resorption [6, 18, 19]. The remedies contain placenta which has been prepared as a hot water extract for use in Chinese medicine. Since the availability of *Hominis* placenta is limited, porcine placenta has also been used. The ovarian hormones and amino acid profiles of porcine placenta are similar to those of *Hominis* placenta [20]. However, the effects and mechanisms underlying the placenta-associated health benefits remain poorly characterized and its active components have not been identified. Since the suppression of bone resorption is associated with Wnt/*β*-catenin signaling, this signaling was studied to determine the anti-osteoporosis mechanism of porcine placenta hydrolysates [11]. 

Among the major active components in placenta are ovarian hormones such as estrogen and progesterone, since estrogen plays a positive role in bone biology and osteoporosis prevention and treatment, primarily by decreasing bone resorption [9, 21, 22]. The active compounds in the placenta are likely water soluble (such as amino acids) since the remedies are prepared by boiling in water. However, estrogen and progesterone are not extractable by hot water. Thus, the positive effect of remedies such as *Hominis* placenta may not be due to estrogen and progesterone, but due to the profiles of amino acids and modified amino acids. It is therefore important to study the anti-osteoporotic activity of porcine placenta hydrolysates with and without removing ovarian hormones by modifying the filtering system to change the contents of lipids and amino acids in placenta hydrolysates. In the present study, the anti-osteoporotic effects of porcine placenta hydrolysates with and without ovarian hormones were examined in ovariectomized (OVX) rats fed calcium-deficient diets. 

## 2. Materials and Methods

### 2.1. Production and Composition of Porcine Placenta Hydrolysates

Porcine placenta hydrolysates were prepared following the procedures of Codebio Inc. (Cheonan, Republic of Korea). Placentas were first thawed using a defroster and washed with saline at 16°C to remove blood and cords. They were then hydrolyzed using papain, bromelain, pronase, and Alcalase at 70 ± 0.1°C for 48 h (pH 4.5–6.0). Next, the protein hydrolysis enzymes were inactivated at 100 ± 0.1°C for 30 min and the hydrolysates were filtered through activated carbon. The lipids were then removed by mixing with calcium and phosphate salts and by filtering through two types of absorbents. Finally, the hydrolysates were adjusted to pH 7.0 ± 0.2 using calcium and phosphate salts. The 17*β*-estradiol and progesterone contents of the hydrolysates were measured using an ELISA kit, according to the manufacturer's instructions (Abcam, Cambridge, MA, USA). Hydrolysates with both various ovarian hormone contents and with no hormones were prepared. 

 WPPH and NPPH were mixed with AccQ-Fluor buffer and Acc-Fluor reagent (Waters Corporation, Milford, MA, USA), and the mixture was heated at 80°C. An aliquot of the sample was passed through a 0.45 *μ*m filter and injected into the chromatographic system. Chromatographic separations were performed on an AccQ-Tag column (150 × 2.1 mm, 3 *μ*m particle size; Waters Corporation) in an HPLC instrument. A gradient mixture of AccQ-Tag Eluent (A) and acetonitrile (B) was used as the mobile phase at a flow rate of 1 mL/min at 30°C. The eluent composition started with 100% A and was linearly decreased to 67% over 33 min. It was switched to 100% B for 3 min and then switched back to 100% A for 26 min. Amino acids were detected using a fluorescence detector at excitation and emission wavelengths of 250 and 395 nm, respectively. A standard solution containing 22 amino acids at concentrations of 0.25–10 mg/L was prepared with 0.1 M HCl. Six-point calibration curves were generated by plotting the peak area *versus* concentration for each amino acid. 

### 2.2. Animals

 Female Sprague-Dawley rats (weighing 227 ± 18 g) were used. The animals were housed individually in stainless steel cages in a controlled environment (23°C and with a 12 h light/dark cycle). All surgical and experimental procedures were performed according to the guidelines of the Animal Care and Use Review Committee of the Hoseo University, Republic of Korea (2011-2012). Since bone formation and resorption are slow processes, the experiment needed to proceed for at least 12 weeks and a calcium-deficient diet was provided to accelerate bone loss [15–17]. Experimental animals were provided water and calcium-deficient diets *ad libitum* during the 12-week experimental period. The calcium-deficient diet was prepared by a semipurified method with a modified AIN-93 formulation for experimental animals [23]. The diet consisted of 40 energy percent (En%) carbohydrates, 20 En% protein, 40 En% fats, and 1.7 g calcium/kg diet (one-third of the AIN-93 formulation). The major carbohydrate, protein, and fat sources were starch plus sugar, casein (milk protein), and lard (CJ Co, Seoul, Republic of Korea), respectively. 

### 2.3. Experimental Design

 Rats underwent ovariectomy or a sham operation under anesthesia by intramuscular injection of a ketamine and xylazine mixture (100 and 10 mg, resp.). Forty OVX rats were randomly assigned to the following four groups: (1) porcine placenta hydrolytes with ovarian hormones (WPPH), (2) porcine placenta hydrolytes without ovarian hormones (NPPH), (3) estrogen replacement (EST), and (4) dextrose (placebo; OVX-control). Ten sham-operated (Sham) rats were assigned to the placebo group (Sham-control). Our preliminary study found that the original placenta hydrolytes increased femur and spine BMD in a dose-dependent manner and that daily administration of 1,000 mg/kg bw/day original placenta hydrolysates improved the bone-mass density of OVX rats. In the present study, OVX rats in the appropriate groups were orally administered 1,000 mg/kg bw/day of porcine placenta hydrolysates with or without ovarian hormones or dextrose, while OVX rats in the EST group orally received 0.1 mg/kg bw conjugated estrogen (Dalim Biotech, Repulic of Korea). Sham rats were orally provided 1,000 mg/kg bw/day dextrose as a normal control. Food and water intake, as well as body weight, were measured every Tuesday at 10 AM. One day before being euthanized, urine samples were collected from the rats, which were then housed individually for 24 h in metabolic cages. At the end of the study, rats were anesthetized with ketamine and xylazine (100 and 10 mg/kg body weight, resp.). Peri-uterine and retroperitoneal fat mass and uteruses were then removed and weighed. Uterus index was calculated as uterus weight divided by body weight. Blood samples for serum isolation were collected by abdominal cardiac puncture. Urine and serum samples were then stored at −70°C for biochemical analysis. 

### 2.4. BMD Measurement

 After anesthetization with ketamine and xylazine (100 and 10 mg/kg bw, resp.), BMDs of the right femur and lumbar spine were measured using a dual-energy X-ray absorptiometer (Norland pDEXA Sabre; Norland Medical Systems Inc., Fort Atkinson, WI, USA), which was equipped with the appropriate software for assessment of bone density in small animals. 

### 2.5. Assay for Serum and Urine Chemistry

Serum calcium (Ca), serum phosphorus (P), serum alkaline phosphatase (ALP), urine Ca, urine P, and urine creatinine levels were measured by standard colorimetric methods using commercial kits (Asan, Seoul, Republic of Korea). Serum osteocalcin (Osteocalc in-EIA, Biomedical Technology Inc., USA) and bone-specific alkaline phosphatase (BSALP) and urinary deoxypyridinoline (DPD; METRATM BAP EIA kit, Quidel Corp.) concentrations were determined by ELISA using the respective kits according to the manufacturer's instructions.

### 2.6. Isolation of Total Femur RNA and Real-Time PCR

Rat femurs were collected at the end of treatment. Femurs were individually powdered with a cold steel mortar and pestle and then mixed with a monophasic solution of phenol and guanidine isothiocyanate (TRIzol reagent, Gibco-BRL, Rockville, MD, USA) for total RNA extraction, following the manufacturer's instructions. cDNA was synthesized from equal amounts of total RNA using superscript III reverse transcriptase, and polymerase chain reaction (PCR) was performed with high-fidelity Taq DNA polymerase. Equal amounts of cDNA were added to SYBR Green mix (Bio-Rad, Richmond, CA, USA) and amplified using a real-time PCR instrument (Bio-Rad). The expression levels of the genes of interest were normalized to that of the housekeeping gene, *β*-actin. To assess changes in the expression of Wnt pathway genes, the following primers were used: GGCTCGGATGAAGCTAACTG (forward) and CAGGATGATGCCAATGACAG (reverse) for low-density lipoprotein receptor-related protein 5 (LRP5), GCTGCATGAGGCACGCTAT (forward) and GGGCATGCATATTCCGTTT (reverse) for dickkopf-related protein 1 (DKK1), GGAAAGCAAGCTCATCATTCT (forward) and AGTGCCTGCATCCCACCA (reverse) for *β*-catenin, GCTCGAAAGTACAGGAACAGA (forward) and GCCGAGGAAGGGAGAGAACGAT (reverse) for RANKL, and AGCGTGGCTACAGCTTCACC (forward) and AAGTCTAGGGCAACATAGCACAGC (reverse) for *β*-actin. At least four PCR reactions per group were performed. 

### 2.7. Statistical Analysis

Statistical analysis was performed using SAS, version 7.0. Results are expressed as means ± standard deviations. The anti-osteoporotic effects of NPPH, WPPH, and EST were compared by one-way analysis of variance. Significant differences between groups were identified by Tukey's tests. The OVX and Sham groups were compared using two-sampled *t*-tests. A value of *P* < 0.05 was considered to indicate statistical significance.

## 3. Results

### 3.1. Composition of Porcine Placenta Hydrolysates

 Ovarian hormones and amino acid profiles were altered by removal of lipids by filtration. NPPH contained negligible levels of ovarian hormones, while WPPH contained substantial amounts of estrogen and progesterone due to incomplete lipid elimination (Table 1). The amino acid profiles of NPPH and WPPH also differed (Table 1). The total amino acid level of NPPH was lower than that of WPPH but contained a greater proportion of essential amino acids. Thus, some amino acids may have existed in modified forms, such as hydroxyproline and hydroxylysine.

### 3.2. Body and Organ Weights

 After 12 weeks, body weights and peri-uterine fat and retroperitoneal fat masses were higher in OVX rats than in Sham rats. NPPH inhibited the increment of OVX-induced weight gain, but the inhibition was less than that of estrogen. WPPH had no effect on body weight and fat mass increases (Table 2). OVX caused significant atrophy of uterine tissue compared to the Sham group (*P* < 0.01), and NPPH significantly increased both absolute and relative uterine weights (Table 2). EST had no significant effect. As expected, serum 17*β*-estradiol levels were significantly reduced in OVX rats but were unaffected by NPPH or WPPH. However, EST increased 17*β*-estradiol levels compared to those in Sham rats.

### 3.3. Bone Mineral Density

**Figure 1:**
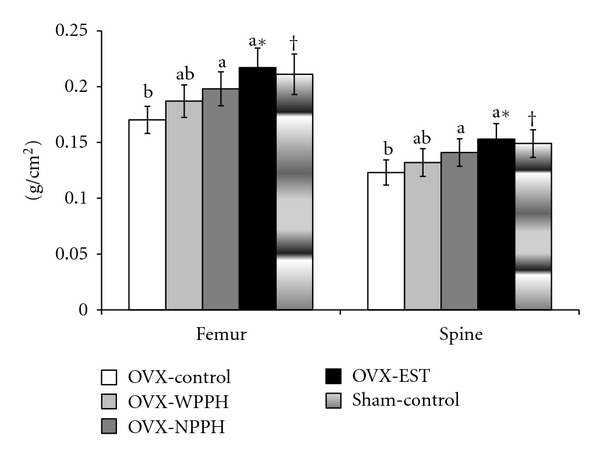
Total bone mineral densities (BMDs) in the femur and lumbar spine at the end of the study period. Ovariectomized (OVX) rats were divided into four groups, and for 12 weeks each group was orally administered 1 g/kg bw/day of porcine placenta hydrolysates with (WPPH) or without ovarian hormones (NPPH), 0.1 mg/kg bw/day conjugated estrogen (EST), or dextrose (OVX-control), while Sham rats were orally administered 1 g/kg/day dextrose as a normal control (Sham-control). At the end of the experimental period, BMDs of the femur and lumbar spine were measured by DXA. Values are expressed as means ± SD (*n* = 10). *Significantly different among the treatments at *P* < 0.05. ^a, b, c^Significantly different among the OVX groups by Tukey's test at *P* < 0.05. ^†^Significantly different between the OVX-control and Sham-control groups at *P* < 0.05.

Right femur and lumbar spine BMDs in OVX rats were decreased by 18.6 and 15.8%, respectively (*P* < 0.01), compared with Sham rats at the end of the 12-week experimental period (Figure 1). The NPPH group exhibited significant 18.7 and 17.9% increases in the BMDs of the right femur and lumbar vertebra, respectively, compared to the OVX-control group (Figure 1). However, these increases were lower than that elicited by estrogen treatment. Generally, WPPH elevated BMD, but the effects were not significant. Estrogen treatment ameliorated the OVX-mediated reduction of right femur and lumbar spine BMD, returning the levels to the baseline (Sham group). Thus, NPPH treatment prevented the reduction in BMD; however, estrogen did not play a role since serum estrogen levels of NPPH-treated rats were similar to those of the OVX-control rats.

### 3.4. Serum and Urinary Ca and P Levels

 OVX rats exhibited significantly increased serum P levels and increased urinary Ca and P levels compared to Sham rats (Table 3). NPPH administration reversed the OVX-induced changes in serum P levels and urinary Ca and P levels (*P* < 0.01), similar to EST-treated rats. However, serum Ca levels were not significantly altered by OVX, NPPH, or EST (Table 3).

### 3.5. Bone Turnover Markers

 The OVX operation significantly increased serum osteocalcin, ALP, and BSALP concentrations by 44.3, 38.7, and 24.5%, respectively, compared to the Sham-operated rats (Table 4). There were no significant differences in the levels of WPPH-treated rats compared to the OVX-control rats. NPPH treatment of OVX rats significantly decreased serum osteocalcin, ALP, and BSALP concentrations in comparison to the OVX-control group (Table 4). However, this decrease was not as significant as that induced by estrogen (Table 4). 

### 3.6. Gene Expression Involved in Wnt Signaling in the Femur

**Figure 2:**
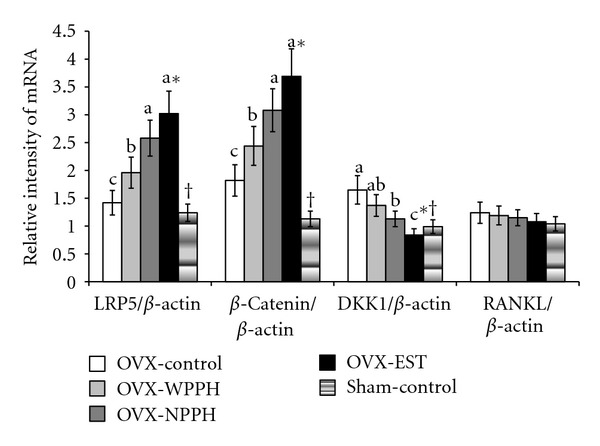
Femur relative mRNA levels of the Wnt signaling proteins; LRP5, *β*-catenin, DKK1, and RANKL. Ovariectomized (OVX) rats were divided into four groups, and for 12 weeks each group was orally administered 1 g/kg bw/day of porcine placenta hydrolysates with (WPPH) or without ovarian hormones (NPPH), 0.1 mg/kg bw/day conjugated estrogen (EST), or dextrose (OVX-control), while Sham rats were orally administered 1 g/kg/day dextrose as a normal control (Sham-control). After the experimental period, mRNA levels in the femur were measured by real-time PCR. Values are expressed as means ± SD (*n* = 5). *Significantly different among the treatments at *P* < 0.05.   ^a, b, c^Significantly different among the OVX groups by Tukey's test at *P* < 0.05. ^†^Significantly different between the OVX-control and Sham-control groups at *P* < 0.05.

To determine the expression levels of the Wnt pathway genes, LRP5, *β*-catenin, DKK1, and RANKL mRNA levels were monitored. *β*-catenin and DKK1 mRNA levels in the femur were upregulated in the OVX-control group compared to the Sham-control group (Figure 2). However, LRP5 and RANKL mRNA levels in OVX rats were not significantly altered. LRP5 and *β*-catenin mRNA levels were greater in NPPH-treated OVX rats than that in the OVX-control rats and were further increased in OVX rats by estrogen treatment (Figure 2). However, DKK1 expression was decreased by NPPH and estrogen, while RANKL expression was not affected by any treatment.

## 4. Discussion

The regulation of bone mass is an active and dynamic process orchestrated by bone-forming osteoblasts and bone-resorbing osteoclasts [1, 2]. Osteoporosis, the most common bone remodeling disease, is caused mainly by an increase in bone resorption, which is not compensated for by a similar increase in bone formation [1, 2]. Postmenopausal osteoporosis is characterized by bone fragility due to an imbalance between osteoclast-mediated bone resorption and osteoblast-mediated bone formation [24]. Estrogen plays a crucial role in the regulation of bone mass by maintaining the balance between bone resorption and formation [4, 24]. Hormone replacement therapy can prevent or alleviate post-menopausal osteoporosis but has adverse side effects such as cancer [4]. 

The OVX rat model has many similarities to actual bone loss in humans [8, 14] and has been widely used to evaluate potential treatments for osteoporosis. Therefore, we investigated the anti-osteoporotic effect of PPH with and without ovarian hormones in OVX rats. The 12-week treatment with NPPH prevented reduction of the BMD of the femur and spine by reducing urinary excretion of Ca and P and bone turnover by decreasing Wnt signaling in OVX rats. WPPH exhibited similar effects; however, these were not significant. This suggests that the anti-osteoporotic effect of NPPH was not due to estrogen, since NPPH did not contain 17*β*-estradiol. Thus, the active compounds may include typical or modified amino acids.

Serum Ca and P levels play important roles in the release of calcitonin and PTH from the thyroid and parathyroid glands, respectively [25]. These hormones regulate serum Ca levels within the normal range by modulating Ca accumulation in the bone, reabsorption from kidney tubules and absorption from the intestines [25, 26]. OVX rats exhibit higher serum P levels and urinary Ca and P levels due to increased bone resorption, but their serum Ca levels remain unclear [15, 16]. Cheng et al. [14] demonstrated that Er-Zhi-Wan, a Chinese herbal remedy, reduces the OVX-induced increased rates of urinary Ca and P excretion. Our findings indicate that serum Ca levels were increased in OVX rats compared to Sham rats, but the difference was not significant. None of the treatments affected serum Ca levels in OVX rats. However, serum P levels and urinary Ca and P levels were higher in OVX rats than Sham rats and were significantly decreased after treatment with NPPH and EST. Thus, our data suggest that the bone turnover rate was downregulated by NPPH.

 Serum ALP, BALP, and osteocalcin, and urinary DPD, levels are widely accepted as bone turnover markers [27–29]. Osteocalcin is an abundant noncollagenous protein in the bone matrix, and the osteocalcin content of bone is an important bone growth marker [27]. BSALP is a glycoprotein ectoenzyme linked to the osteoblast membrane [28]. Osteocalcin and BSALP are released into the circulation during the bone remodeling process and during pathophysiological states. Bones are the main contributors to BALP and osteocalcin expression [27, 28]. In addition, DPD and pyridinoline are found in osteoclasts and are released and excreted into urine upon loss of bone cells [17, 29]. Therefore, serum BSALP and osteocalcin, as well as urinary DPD, are useful markers of bone turnover. The present study showed that 12-week treatment with NPPH in OVX rats significantly increased serum BALP and osteocalcin levels, and urinary DPD levels. These results are consistent with the report by Chae et al. [8] that *Hominis* placenta extract reduced serum P, ALP, and thyroxine levels and decreased bone loss in OVX rats. 

In previous studies, after ovariectomy, rat BMD was markedly decreased compared to that of Sham-operated rats, due to an increase in bone turnover [13–15]. Estrogen is known to regulate bone remodeling before menopause in women [30, 31], and estrogen deficiency has been reported to accelerate bone loss [32]. Estrogen replacement therapy is used to alleviate post-menopausal osteoporosis via estrogen receptor activation to enhance osteogenic properties [9, 21]. However, the adverse effects of long-term estrogen usage (such as breast cancer and cardiovascular disease) reduce the popularity of this treatment [21, 22]. Our findings indicate that OVX rats had significantly decreased femur and lumbar spine BMDs, and after 12 weeks of NPPH treatment, BMD loss in the femur and lumbar vertebra was significantly inhibited in OVX rats with no increase in serum estrogen levels. This inhibition was less marked than that caused by estrogen treatment; however, the estrogen-mediated inhibition was not significant (*P* = 0.09). Since the experimental period was relatively short, this effect may be significant over longer periods of time. Considering the adverse effects of estrogen replacement, NPPH might be sufficient to prevent post-menopausal bone loss. Consistent with the present study, *Hominis *placenta inhibited the BMD decrease in OVX rats [8].

A Few studies have examined the placenta itself [8, 33]. A recent study by Takuma et al. [33] has demonstrated that porcine placenta has not anti-osteoporotic activity in mice, but this study has some limitations for determining the prevention of osteoporosis due to a short-term treatment (5 weeks) and a diet containing sufficient Ca. However, some remedies that contain *Hominis* placenta, such as Yukmi-Jihangtang-Jahage, Saenghyuldan, and Honghwain-Jahage, are known to prevent bone resorption through their anti-inflammatory activities [6, 18, 34]. Cytokines in bone play a crucial role in the pathogenesis of osteoporosis. In estrogen-deprived bone cells, levels of cytokines such as interleukin-1*β* (IL-1*β*), tumor necrosis factor-*α* (TNF-*α*), and/or interleukin-6 (IL-6) are elevated and may be responsible for bone loss [35]. In addition, receptor activator of nuclear factor-*κ*B ligand (RANKL) has been identified as the major cytokine in osteoblast and osteoclast communication [36]. Yukmi-Jihangtang-Jahage inhibited COX-2 expression and suppressed IL-1*β*, TNF-*α*, and IL-6 levels in mouse calvarial osteoblasts, which may decrease bone resorption [18]. In addition, Yukmi-Jihangtang-Jahage decreased bone resorption in OVX rats. Similarly, Honghwain-Jahage inhibited IL-1*β*-induced bone resorption in cultured osteoblast cells derived from mouse calvarial bone explants [6]. Thus, placenta-containing remedies prevented bone loss by decreasing its resorption via suppression of inflammatory processes. However, RANKL expression was not significantly reduced by NPPH, WPPH, or estrogen replacement in the present study. Thus, the major pathway of NPPH-mediated bone loss prevention may not involve alleviation of inflammation by reducing cytokine concentrations. 

Proteins in the Wnt signaling pathway are regulators of bone mass [37, 38]. Canonical Wnt signaling can regulate osteoblast function, contributing to bone formation [39, 40]. Wnt signaling is initiated by binding of a Wnt family member to two receptor molecules, namely, Frizzled proteins and LRP5/6. This stabilizes cytoplasmic *β*-catenin, after which the accumulated *β*-catenin is translocated into the nucleus where it stimulates Wnt target genes, including c-myc, Runx2, and cyclin D1 [41]. Activation of the Wnt/*β*-catenin signaling pathway promotes proliferation and differentiation of osteoblast precursor cells and increases osteoblast activity, which favors the deposition of new bone and increases BMD [41]. DKK1 negatively regulates Wnt/*β*-catenin signaling by binding to and antagonizing the Wnt coreceptors Lrp5/6 [42]. In the present study, the expression of proteins involved in Wnt signaling was elevated in OVX compared to Sham rats; however, NPPH and estrogen treatment increased these to levels higher than those of OVX-control rats. Thus, OVX-induced osteogenesis can be used to overcome osteoclast action but may not in itself be sufficient. NPPH and estrogen treatments stimulate Wnt signaling, thus overcoming osteoclast activity. In addition, the expression of DKK1, a negative regulator of Wnt signaling, was markedly increased in OVX rats and inhibited the stimulation of Wnt signaling. Also, NPPH and estrogen treatment suppressed DKK1 expression and activated Wnt signaling. Thus, NPPH and estrogen stimulated Wnt signaling in OVX rats, resulting in increased osteogenesis. 

The limitation of this study was that the active component in NPPH was not identified. Since NPPH does not include ovarian hormones, its anti-osteoporotic activity is not associated with ovarian hormones, such as estrogen. WHHP had a lesser effect on anti-osteoporotic activity, which may be related to the low levels of ovarian hormones it contains. Thus, improvement of the amino acid profiles or the presence of modified amino acids in NPPH should be considered. Nutrients and trace minerals, including essential amino acids, such as arginine, lysine, vitamin K, Mn, B, vitamin D, Zn, Cu, folate, and Si, are often used to improve bone structure [43]. Several studies suggest that dietary arginine and lysine may play an important role in the development, growth, and modeling of long bones [44–46]. Arginine is involved both in the synthesis of substrates (polyamine and L-Pro) implicated in collagen synthesis and in the production of growth hormone, insulin-like growth factor-I, and nitric oxide [46]. In addition, arginine is thought to alleviate metabolic disturbances in Ca absorption, growth, dentition and ossification defects, rachitism, osteomalacia, decalcification, and convalescence [47]. NPPH contained large amounts of arginine and essential amino acids (more than WPPH). Thus, arginine and other essential amino acids may be used to treat osteoporosis.

## 5. Conclusions

NPPH prevented BMD loss in the femur and lumbar spine of OVX rats compared to the OVX-control, but the effect was less marked than that mediated by estrogen treatment. However, WPPH had less of an effect than NPPH. The NPPH-mediated improvement in BMD was associated with reduced bone resorption, as indicated by the decreased serum osteocalcin and BSALP levels, bone turnover markers, and lower serum P levels and urinary Ca and P excretion. The increase in BMD induced by EST was low but more marked than that induced by WPPH. NPPH and estrogen upregulated the expression of proteins (LRP5 and *β*-catenin) involved in Wnt signaling in OVX rats, while that of DKK1, an antagonist of Wnt signaling, was suppressed. Further studies are needed to confirm which component has anti-osteoporotic activity and to study its safety and efficacy for treating osteoporosis. In conclusion, NPPH had an antiosteoporotic effect, activating osteogenesis by stimulating Wnt signaling. This effect may have been mediated by amino acids but was not due to ovarian hormones. Thus, NPPH may represent an alternative treatment for postmenopausal osteoporosis.

## Figures and Tables

**Table 1:** Ovarian hormones and amino acid compositions of porcine placenta hydrolysates.

	WPPH	NPPH
Estrogen (ng/g)	7.24 ± 0.79	0.006 ± 0.001***
Progesterone (ng/g)	74.3 ± 7.52	0.071 ± 0.001***

Aspartate	0.115 ± 0.011	0.082 ± 0.012*
Threonine	0.136 ± 0.014	0.109 ± 0.022
Serine	0.184 ± 0.020	0.133 ± 0.012*
Glutamate	0.295 ± 0.022	0.207 ± 0.021*
Glycine	0.274 ± 0.032	0.131 ± 0.018*
Alanine	0.284 ± 0.018	0.186 ± 0.019*
Valine	0.205 ± 0.028	0.203 ± 0.029
Cysteine	0.062 ± 0.005	0.033 ± 0.001**
Methionine	0.087 ± 0.092	0.126 ± 0.138*
Isoleucine	0.154 ± 0.018	0.189 ± 0.024*
Leucine	0.187 ± 0.020	0.168 ± 0.015
Tyrosine	0.173 ± 0.017	0.098 ± 0.013*
Phenylalanine	0.121 ± 0.018	0.161 ± 0.021*
Lysine	0.224 ± 0.031	0.339 ± 0.037**
Histidine	0.031 ± 0.008	0.063 ± 0.077*
Arginine	0.087 ± 0.018	0.181 ± 0.018**
Proline	0.031 ± 0.003	0.087 ± 0.021**
Total amino acids	2.630 ± 0.255	2.476 ± 0.267
Essential amino acids	1.155 ± 0.121	1.358 ± 0.144*
% Essential amino acids	43.9 ± 0.45	55.9 ± 0.58**

Values are mean ± SD (*n* = 3). Unit of amino acids is mg/mL in 25% porcine placenta hydrolysate.

*Significantly different from WPPH at *P* < 0.05, **P < 0.01, ***P < 0.001.

**Table 2:** Body weights, peri-uterine and retroperitoneal fat weights, uterus weights, and serum 17*β*-estradiol levels at the end of the 12-week study period.

	OVX-control	OVX-WPPH	OVX-NPPH	OVX-EST	Sham-control
	(*n* = 10)	(*n* = 10)	(*n* = 10)	(*n* = 10)	(*n* = 10)
Body weight (g)	329 ± 25^a^	313 ± 24^ab^	297 ± 23^b^	274 ± 25^c∗^	271 ± 23^†^
Peri-uterine fat (g)	6.4 ± 0.8^a^	5.8 ± 0.7^ab^	5.2 ± 0.7^b^	4.3 ± 0.5^c∗^	3.9 ± 0.5^††^
Retroperitoneum fat (g)	8.8 ± 1.0^a^	8.1 ± 0.9^ab^	7.2 ± 0.8^b^	6.1 ± 0.7^c∗^	5.4 ± 0.7^††^
Uterus weight (g)	1.3 ± 0.2^b^	1.4 ± 0.2^ab^	1.6 ± 0.3^a^	1.8 ± 0.3^a∗^	1.9 ± 0.3^†^
Uterus index (mg/g)	4.0 ± 0.5^b^	4.5 ± 0.6^ab^	5.5 ± 0.8^b^	6.5 ± 0.9^c∗^	7.0 ± 0.9^††^
Serum 17*β*-estradiol (pg/mL)	9.6 ± 1.1	9.8 ± 1.0	10.8 ± 1.3	24.8 ± 3.9^a∗^	20.3 ± 3.2^†††^

Uterus index was calculated as uterus weight divided by body weight.

Values are mean ± SD. *Significantly different among the different treatments in OVX rats at *P* < 0.05. ^a,b,c^Values with different superscripts were significantly different among the OVX groups by Tukey's test at *P* < 0.05. ^†^Significantly different between OVX-control and Sham-control at *P* < 0.05, ^††^ *P* < 0.01, ^†††^ *P* < 0.001.

**Table 3:** Serum and urinary Ca and P levels at the end of the 12-week treatment.

	OVX-control	OVX-WPPH	OVX-NPPH	OVX-EST	Sham-control
	(*n* = 10)	(*n* = 10)	(*n* = 10)	(*n* = 10)	(*n* = 10)
Serum calcium (mg/dL)	10.9 ± 1.3	10.6 ± 1.4	10.2 ± 1.1	10.1 ± 1.2	10.1 ± 1.3
Serum phosphorus (mg/dL)	8.18 ± 1.02^a^	7.65 ± 0.92^ab^	7.07 ± 0.91^b^	6.97 ± 0.93^b∗^	6.94 ± 1.12^†^
Urinary calcium (mg/g creatinine)	1.32 ± 0.27^a^	1.12 ± 0.21^b^	0.91 ± 0.18^c^	0.81 ± 0.16^c∗^	0.86 ± 0.14^†^
Urinary phosphorus (mg/g creatinine)	3.87 ± 0.71^a^	3.18 ± 0.64^ab^	2.84 ± 0.45^c^	2.62 ± 0.46^c∗^	2.25 ± 0.38^†^

Values are mean ± SD. *Significantly different among the different treatments in OVX rats at *P* < 0.05. ^a,b,c^Values with different superscripts were significantly different among the OVX groups by Tukey's test at *P* < 0.05. ^†^Significantly different between OVX-control and Sham-control at *P* < 0.05.

**Table 4:** Bone turnover marker levels at the end of the 12-week treatment.

	OVX-control	OVX-WPPH	OVX-NPPH	OVX-EST	Sham-control
	(*n* = 10)	(*n* = 10)	(*n* = 10)	(*n* = 10)	(*n* = 10)
Serum osteocalcin (ng/mL)	8.8 ± 1.1^a^	7.9 ± 1.0^ab^	7.2 ± 0.9^b^	6.3 ± 0.8^c∗^	6.1 ± 0.6^†^
Serum ALP (IU/L)	197 ± 27.8^a^	174 ± 28.4^ab^	153 ± 26.7^b^	135 ± 18.9^c∗^	142 ± 17.5^†^
Serum BSALP (IU/L)	19.3 ± 2.6^a^	18.0 ± 2.3^ab^	16.8 ± 2.0^b^	15.7 ± 1.9^b∗^	15.5 ± 2.1^†^
Urinary DPD (nmol/mmol creatinine)	38.7 ± 4.2^a^	33.7 ± 4.1^ab^	29.2 ± 3.8^b^	23.4 ± 3.1^c∗^	24.7 ± 3.5^††^

ALP: alkaline phosphatase; BSALP: bone-specific alkaline phosphatase; DPD: deoxypyridinoline. Values are mean ± SD. *Significantly different among the different treatments in OVX rats at *P* < 0.05. ^a,b,c^Values with different superscripts were significantly different among the OVX groups by Tukey's test at *P* < 0.05. ^†^Significantly different between OVX-control and Sham-control at *P* < 0.05, ^††^ *P* < 0.01.
